# Demyelinating sentinel lesion preceding a primary central nervous system lymphoma

**DOI:** 10.1590/0004-282X-ANP-2021-0274

**Published:** 2021-11-30

**Authors:** Flávia Sprenger, Thais Bianco, Bernardo Corrêa de Almeida Teixeira

**Affiliations:** 1 Universidade Federal do Paraná, Hospital de Clínicas, Departamento de Radiologia, Curitiba PR, Brazil. Universidade Federal do Paraná Hospital de Clínicas Departamento de Radiologia Curitiba PR Brazil

**Figure 1.**
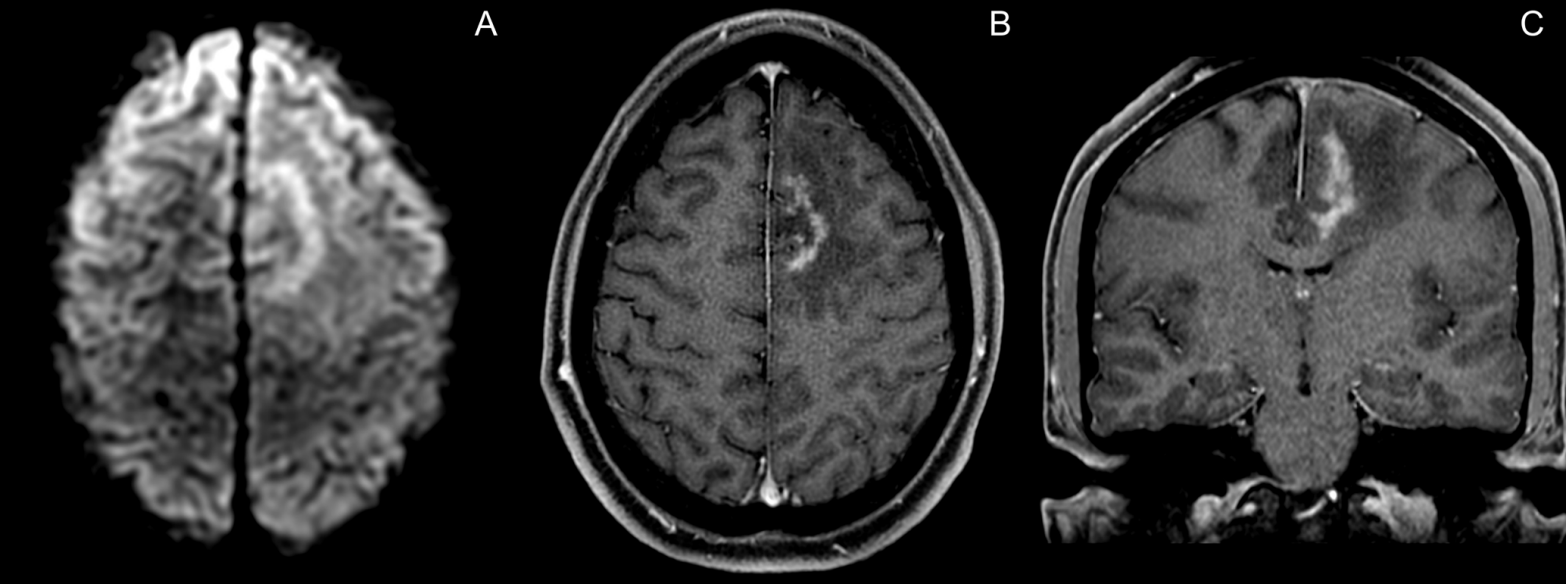
A: Axial diffusion weighted imaging (DWI), showing a left frontal lesion with restricted diffusion on the lesion’s free margin, oriented towards the white matter, suggestive of demyelinating nature. B: Axial post-gadolinium T1, showing contrast enhancement on the lesions free margin. C: Coronal post-gadolinium T1 shows the left frontal lesion, insinuating towards the corpus callosum, but with no frank signs of invasion. Notice the spared brainstem.

**Figure 2.**
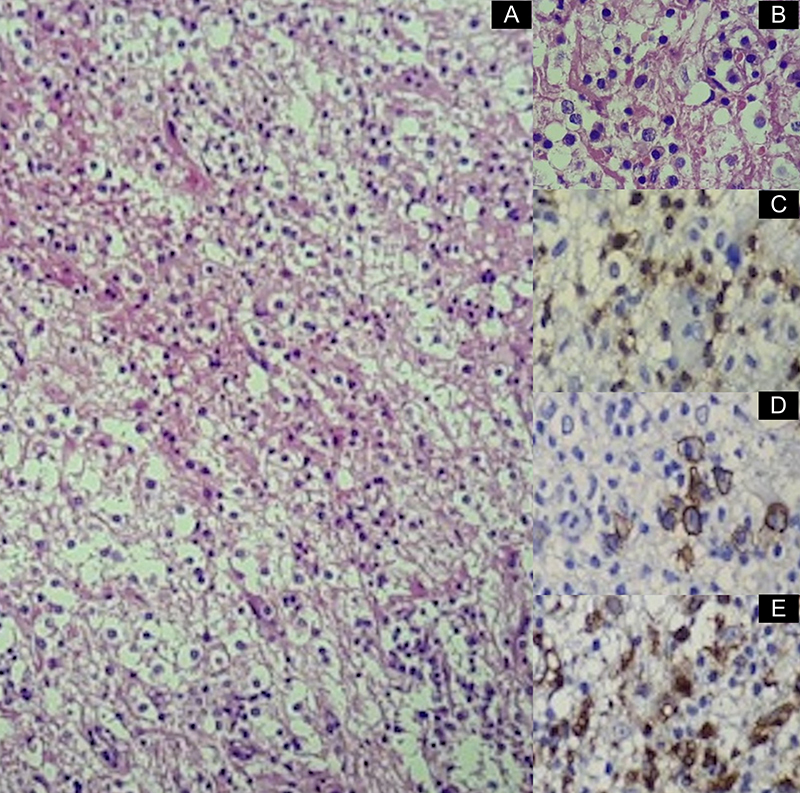
Histopathological findings from surgical biopsy. A: Hematoxicilin-eosin 100x, amplified on B, shows a diffuse inflammatory infiltrate composed by T-lymphocytes, confirmed by immunohistochemistry for CD3 marker on C, plasmacytes (CD138 on D) and foamy macrophages (CD68 on E). The sample was negative for malignancy and markers for B cells were negative (not shown).

**Figure 3.**
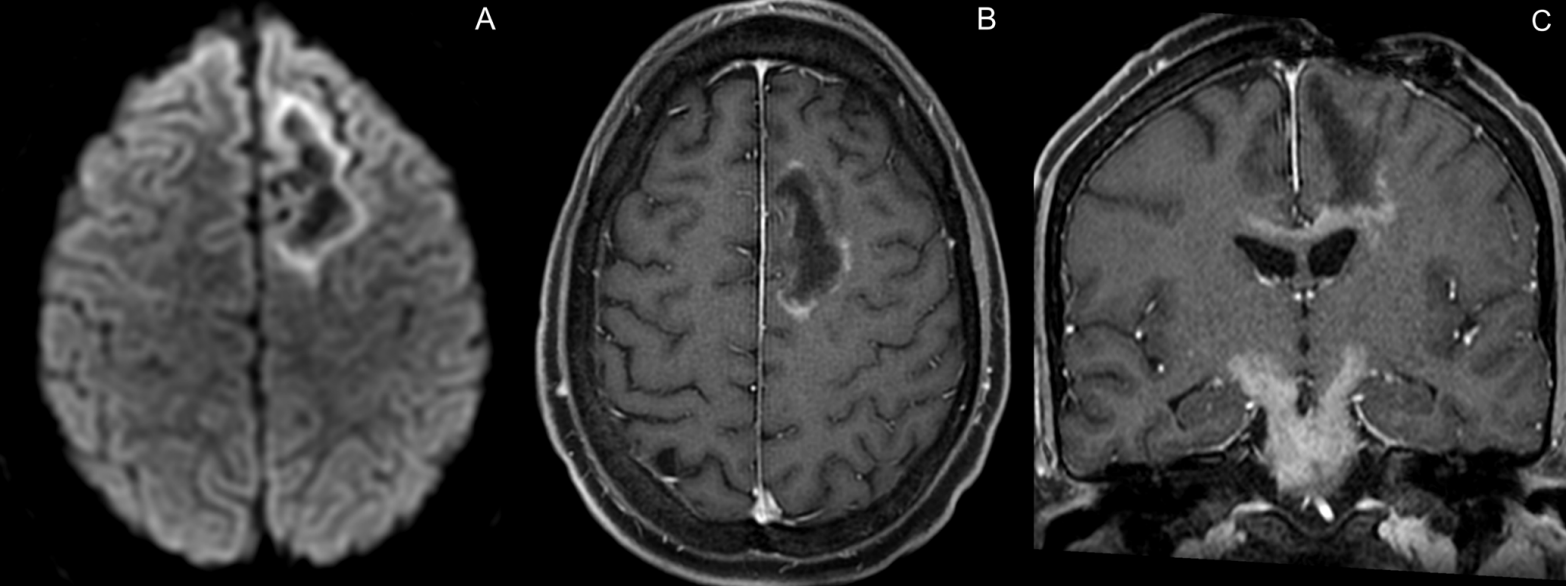
Control MRI two months later, demonstrates persistent restricted diffusion (A), but less enhancement of the left frontal lesion (B). C (coronal post-gadolinium T1): Its caudal aspect extends and invades the corpus callosum. Notice the development of a new and solid-enhancing lesion on the brainstem, extending along the cerebral peduncles and the postoperative changes on the left frontal lobe.

**Figure 4.**
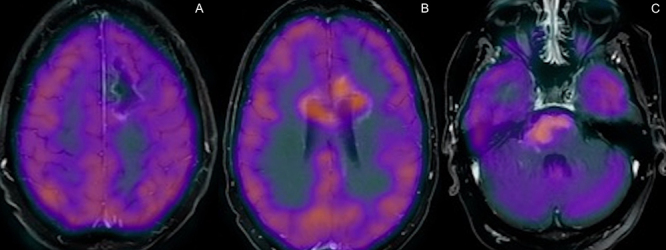
PET-CT and MRI fusion, showing the hypometabolic behavior of the original left frontal lesion (A), in contrast with hypermetabolism on the corpus callosum (B) and brainstem (C) lesions, inferring different etiologies.

A 29-year-old man presented with tonic-clonic seizures. Initial MRI showed a lesion centered on the white matter of the left frontal lobe, with restricted diffusion and contrast enhancement on its margins and low rCBV and hypometabolismon PET-CT, suggestive of a tumefactive demyelination lesion (Figure 1). Patient underwent surgical biopsy, with no signs of malignancy (Figure 2). Two months later, control MRI showed a new lesion on the brainstem, with solid enhancement and hypermetabolism on PET-CT, compatible with lymphoma (Figures 3 and 4). 

Demyelinating sentinel lesions preceding CNS lymphomas are a rare entity and its pathophysiology is not fully understood^1^^,^^2^.
